# Revisiting low-molecular-weight heparin for venous thromboembolism: from pharmacology to precision dosing and implementation

**DOI:** 10.3389/fphar.2026.1824218

**Published:** 2026-06-05

**Authors:** Sha Zhu, Qiang Ni, Ji He

**Affiliations:** 1 Department of Adult Intensive Care Medicine, West China Second University Hospital, Sichuan University, Chengdu, China; 2 Key Laboratory of Birth Defects and Related Diseases of Women and Children (Sichuan University), Ministry of Education, Chengdu, China

**Keywords:** anticoagulant switching, anti–factor Xa monitoring, cancer-associated thrombosis, low-molecular-weight heparin, precision dosing, pregnancy and postpartum, renal impairment, venous thromboembolism

## Abstract

**Background:**

Despite the increasing use of direct oral anticoagulants, low-molecular-weight heparin (LMWH) remains important in venous thromboembolism (VTE) care when oral therapy is unsuitable, temporarily unsafe, or difficult to manage, particularly during pregnancy, cancer-associated thrombosis, renal impairment, and peri-procedural care. Objective: To review how LMWH pharmacology informs practical decisions about dosing, anti-factor Xa monitoring, treatment interruption and restart, and switching between anticoagulants.

**Methods:**

We performed a narrative review of contemporary guidelines, randomized trials, systematic reviews, pharmacokinetic/pharmacodynamic studies, drug labels, and laboratory medicine evidence, focusing on literature published from 1 January 2015 to 18 December 2025, with selected earlier sources retained for foundational pharmacologic principles.

**Results:**

LMWH can be used with standard fixed or weight-based dosing in many stable patients, but reassessment is needed when renal function, body weight, pregnancy physiology, cancer-related bleeding risk, critical illness, or procedural timing changes the relationship between dose and exposure. Anti-factor Xa testing should not be used routinely; it is most useful when sampling is standardized and the result can guide a specific action, such as dose adjustment, interval extension, treatment interruption, or switching to unfractionated heparin.

**Conclusion:**

LMWH remains useful when oral anticoagulants are difficult to use safely. Its clinical value depends on clear dose selection, renal-function reassessment, selective monitoring, planned interruption and restart, and timely switching when patient risk or treatment feasibility changes.

## 1 Introduction

### 1.1 Why LMWH dosing remains clinically important in VTE care

Venous thromboembolism (VTE), including deep vein thrombosis and pulmonary embolism, remains a major cause of preventable morbidity, mortality, long-term disability, and health-system burden worldwide (Khan et al., 2021; Wendelboe and Weitz, 2024; Lutsey et al., 2023). Once VTE is confirmed, therapeutic anticoagulation is usually indicated unless there is active bleeding, urgent surgery, or another major contraindication (Kahn and de Wit, 2022; Freund et al., 2022; Stevens et al., 2021). In contemporary practice, the more difficult decisions often occur after the indication for anticoagulation has been established: which anticoagulant should be selected, whether standard dosing is adequate, when treatment should be interrupted or restarted, and whether monitoring or switching is needed in patients with changing clinical risk (Kahn and de Wit, 2022; Freund et al., 2022; Stevens et al., 2021; Maughan et al., 2025).

Direct oral anticoagulants (DOACs) have simplified VTE treatment for many patients and are now preferred in several guideline-supported settings (Stevens et al., 2021; Maughan et al., 2025). However, low-molecular-weight heparin (LMWH) remains clinically important when oral therapy is unsuitable, temporarily unsafe, insufficiently studied, or operationally fragile. Common examples include pregnancy and the postpartum period, selected patients with cancer-associated thrombosis, patients with unreliable gastrointestinal absorption, clinically important drug–drug interactions, peri-procedural interruption and restart, and patients with severe or fluctuating renal function (Stevens et al., 2021; Maughan et al., 2025; Bistervels et al., 2022; Farge et al., 2022; Lyman et al., 2021). In these settings, the clinical question is not whether LMWH is an older anticoagulant, but how it should be dosed, monitored, held, restarted, or replaced by another anticoagulant.

### 1.2 From predictable pharmacology to patient-specific uncertainty

LMWH is often considered more predictable than unfractionated heparin because of its subcutaneous administration, relatively stable bioavailability, longer half-life, and lower requirement for routine laboratory monitoring (Hogwood et al., 2023; Mulloy et al., 2016). This predictability supports fixed or weight-based dosing in many stable patients. However, it can weaken when drug clearance, distribution, absorption, or bleeding risk changes. Renal impairment may increase LMWH accumulation; extreme body weight may alter exposure; pregnancy changes body weight, plasma volume, and renal clearance; cancer adds both recurrent thrombosis risk and site-specific bleeding risk; and critical illness or peri-procedural care may introduce rapidly changing physiology and repeated interruptions (Stevens et al., 2021; Bistervels et al., 2022; Farge et al., 2022; Hogwood et al., 2023; Mulloy et al., 2016).

Anti-factor Xa (anti-Xa) activity is commonly used when clinicians seek an exposure estimate for LMWH, but it should not be treated as a universal surrogate for clinical efficacy or safety (Nenci, 2003; John et al., 2024). Results depend on sampling time, assay calibration, LMWH product, dose intensity, and clinical context (Hogwood et al., 2023; Mulloy et al., 2016; Nenci, 2003). Therefore, anti-Xa testing is most useful when it answers a specific clinical question and can lead to a defined action, such as dose adjustment, interval extension, switching to unfractionated heparin, or avoiding unnecessary dose changes after a mistimed sample (Nenci, 2003). This distinction is central to precision dosing: monitoring is valuable only when it changes management in a clinically meaningful way.

### 1.3 Aim and scope of this review

This review focuses on practical dosing and monitoring decisions for LMWH in adult VTE prevention and treatment. It does not aim to provide a comprehensive review of VTE diagnosis, all anticoagulant classes, or all prophylaxis strategies. Instead, it addresses four clinically relevant questions:

1. When is standard LMWH dosing likely to be sufficient?
2. When should dose adjustment or closer reassessment be considered?
3. When can anti-factor Xa monitoring provide useful information, and when is it unlikely to change management?
4. When should clinicians consider switching from LMWH to unfractionated heparin, a DOAC, or another strategy?

We discuss these questions in settings where LMWH dosing is most uncertain or clinically consequential: renal impairment, extremes of body weight, pregnancy and postpartum care, cancer-associated thrombosis, critical illness, and peri-procedural management. By linking LMWH pharmacology to bedside decisions, this review aims to provide a clinically usable synthesis for dose selection, selective monitoring, interruption and restart, and switching decisions in complex VTE care.

## 2 Methods and scope

### 2.1 Review design

This article is a narrative, practice-oriented review of LMWH dosing, monitoring, and switching decisions in adult VTE care. The aim was not to perform a quantitative meta-analysis or to produce formal clinical practice recommendations. Instead, we sought to synthesize published evidence and publicly available guidance into clinically usable principles for dose selection, selective anti-factor Xa monitoring, interruption and restart, and transitions between LMWH, unfractionated heparin (UFH), and oral anticoagulants.

All evidence discussed in this review was derived from published studies, published reviews, drug labels, or publicly available clinical guidelines. No unpublished data, original patient-level data, institutional audit data, or non-public clinical pathway data were used.

### 2.2 Search strategy and information sources

We searched MEDLINE via PubMed, Embase, the Cochrane Library, and Web of Science for literature published from 1 January 2015 to 18 December 2025, with the final search performed on 18 December 2025. Reference lists of key guidelines, randomized trials, systematic reviews, and pharmacology reviews were also screened to identify additional relevant sources. Earlier publications were included when they provided foundational pharmacokinetic/pharmacodynamic principles, product-specific LMWH information, or historically important evidence not replaced by more recent data.

The search combined four major concepts: LMWH, VTE, dosing/monitoring, and special populations or clinical contexts. Representative search terms included: “low molecular weight heparin,” “LMWH,” “enoxaparin,” “dalteparin,” “tinzaparin,” “nadroparin,” “venous thromboembolism,” “deep vein thrombosis,” “pulmonary embolism,” “anti-Xa,” “anti-factor Xa,” “dose adjustment,” “pharmacokinetics,” “pharmacodynamics,” “renal impairment,” “obesity,” “low body weight,” “pregnancy,” “postpartum,” “cancer-associated thrombosis,” “critical illness,” “perioperative,” “periprocedural,” “bridging,” “hold,” “restart,” and “switching.” Controlled vocabulary terms, including MeSH and Emtree terms where available, were combined with free-text terms. Detailed search concepts and representative search terms are provided in the Supplementary Methods.

### 2.3 Eligibility and prioritization of evidence

We prioritized clinical practice guidelines, randomized controlled trials, systematic reviews, meta-analyses, pharmacokinetic/pharmacodynamic studies, high-quality observational studies, drug labels, and laboratory medicine reviews when they were relevant to LMWH dosing, monitoring, safety, or switching decisions in adult VTE prevention or treatment. Evidence was prioritized when it addressed one or more of the following questions: standard versus adjusted LMWH dosing; anti-Xa monitoring and sampling; renal impairment and accumulation; extremes of body weight; pregnancy and postpartum management; cancer-associated thrombosis; critical illness; peri-procedural interruption and restart; bleeding management; or transition between LMWH, UFH, DOACs, and vitamin K antagonists.

We excluded studies focused exclusively on non-VTE indications, such as acute coronary syndromes, dialysis-circuit anticoagulation, or extracorporeal circuit anticoagulation, unless the findings were directly relevant to LMWH pharmacology or monitoring principles. Pediatric studies were not routinely included because this review focuses on adult VTE care. Laboratory-only studies were included only when they informed the interpretation or limitations of anti-Xa monitoring. Non-English publications were not routinely included unless they were highly relevant and no comparable English-language source was available.

### 2.4 Data extraction and synthesis

For each included source, we extracted information relevant to clinical decision-making, including population, LMWH product, indication, dose strategy, renal function considerations, body-weight considerations, anti-Xa sampling or target information, bleeding and recurrent VTE outcomes where available, and guidance on interruption, restart, or switching. Because the included evidence covered heterogeneous populations, study designs, LMWH products, dose intensities, and outcomes, quantitative pooling was not performed.

Evidence was synthesized qualitatively using a decision-focused structure. We first summarized pharmacologic principles relevant to LMWH exposure and monitoring. We then mapped these principles to clinical contexts in which standard dosing may be insufficient or where monitoring, dose adjustment, or switching may be considered. Particular attention was given to whether a proposed monitoring or dosing strategy could plausibly change management, rather than only producing a laboratory value.

### 2.5 Scope boundaries

This review focuses on adult VTE prevention and treatment pathways. UFH, DOACs, and vitamin K antagonists are discussed only as comparators or alternatives when relevant to LMWH switching, interruption, restart, or monitoring decisions. The review does not provide a comprehensive guideline for VTE diagnosis, thrombolysis, catheter-directed therapy, pediatric anticoagulation, or non-VTE anticoagulation indications. Proposed tables, figures, and practical frameworks should be interpreted as narrative syntheses of published evidence and publicly available guidance, not as original unpublished clinical data or institution-specific protocols.

## 3 Why LMWH dosing becomes uncertain in selected patients

Standard LMWH regimens are effective and practical for many patients because subcutaneous administration usually produces more predictable anticoagulant exposure than unfractionated heparin and does not require routine laboratory titration (Hogwood et al., 2023; Mulloy et al., 2016). However, this predictability is not absolute. LMWH exposure can change when renal clearance, body weight, subcutaneous absorption, pregnancy physiology, cancer-related bleeding risk, or critical illness alters the relationship between prescribed dose and anticoagulant effect (Stevens et al., 2021; Maughan et al., 2025; Bistervels et al., 2022; Farge et al., 2022; Hogwood et al., 2023; Mulloy et al., 2016).

For this reason, the main dosing problem is not whether LMWH “works” in general, but when standard dosing may become unreliable or unsafe. Renal impairment may lead to accumulation and bleeding, whereas augmented renal clearance in acute illness may contribute to lower exposure (Hogwood et al., 2023; Mulloy et al., 2016; UK Kidney Association Renal Association, 2023; U.S. Food and Drug Administration, 2021). Obesity and very low body weight can create uncertainty about whether weight-based or fixed dosing produces the intended exposure (Hogwood et al., 2023; Nenci, 2003; Appay et al., 2025). Pregnancy and the postpartum period require repeated reassessment because body weight, renal clearance, delivery timing, neuraxial anesthesia, and postpartum bleeding risk change over time (Bistervels et al., 2022; Kang et al., 2024; Royal College of Obstetricians and Gynaecologists RCOG, 2015a; American Society of Hematology, 2025). In cancer-associated thrombosis, anticoagulant choice and dosing are influenced not only by recurrent VTE risk but also by tumor site, mucosal bleeding risk, thrombocytopenia, procedures, and drug–drug interactions (Farge et al., 2022; Kang et al., 2024; Key et al., 2023; Agnelli et al., 2020; Raskob et al., 2018; Young et al., 2018).

These situations define the practical scope of precision dosing in this review. Precision does not mean routine anti-Xa testing or complex dose adjustment for every patient. Rather, it means identifying when standard LMWH dosing is likely to be sufficient, when closer reassessment or selective anti-Xa monitoring may be justified, and when switching to unfractionated heparin, a DOAC, or another strategy may be safer or more feasible. The following sections therefore focus on pharmacologic sources of exposure variability, clinically relevant monitoring, and bedside decisions about dose adjustment, interruption, restart, and switching.

## 4 LMWH pharmacology: from molecule to bedside

### 4.1 Structure origin and the “class effect” fallacy: why LMWHs are not equivalent

LMWHs comprise polydisperse, sulfated polysaccharide chains derived from unfractionated heparin, and their clinical behavior is shaped by product-specific manufacturing processes rather than a uniform “class effect” (Hogwood et al., 2023; Mulloy et al., 2016). Distinct depolymerization chemistries yield characteristic terminal structures, chain-length distributions, and sulfation patterns, which shift mean molecular weight and the proportion of chains containing the antithrombin (AT)-binding pentasaccharide (Hogwood et al., 2023; Mulloy et al., 2016; Nenci, 2003). These structural signatures translate into different anti-Xa/anti-factor IIa (anti-IIa) activity ratios and distinct profiles of nonspecific binding and neutralization; accordingly, enoxaparin, dalteparin, and tinzaparin should be regarded as related products rather than interchangeable molecules (Hogwood et al., 2023; Mulloy et al., 2016; Merli and Groce, 2010; Amerali and Politou, 2022). Across LMWHs, the relationship between milligrams, labeled units, and anti-Xa output is product-specific, reflecting composition and assay calibration (Hogwood et al., 2023; Merli and Groce, 2010). Even “generic” or follow-on preparations require product-specific comparability because small compositional shifts can alter measured activity (Fareed et al., 2004). The bedside implication is practical: dose conversion or therapeutic interchange across LMWHs is not a simple unit-for-unit switch, and anti-Xa interpretation must account for the specific product, assay calibration, and clinical indication (Nenci, 2003; Merli and Groce, 2010).

To operationalize this “class effect” fallacy, Table 1 translates major sources of LMWH non-equivalence into actionable clinical consequences and cautious interchange prompts.

**TABLE 1:** “Class effect fallacy” operational table: where LMWH non-equivalence can create clinical consequences.

Non-equivalence domain	What can differ across LMWHs (mechanistic handle)	Clinical decision impact (what could change)	Practical rule-of-thumb (“cautious interchange” prompts)
Manufacturing + chain distribution	Different manufacturing processes yield heterogeneous molecular-weight distributions and activity profiles	Substitution may alter exposure and safety margins in high-risk phenotypes	Treat substitution as a therapy change in high-stakes contexts; re-confirm dose basis, timing, and monitoring plan
Anti-Xa: anti-IIa activity profile	Relative anti-Xa vs. anti-IIa activity varies across LMWH products	Switching can shift the bleed–thrombosis trade-off when already close to a safety boundary	Avoid assuming equipotency in high bleeding-liability phenotypes; keep the same product through an episode when feasible
Dose units not interchangeable (mg vs. IU)	Labeling conventions and syringe strengths/rounding differ across products	Naïve arithmetic conversion increases under-/overdosing risk	Do not convert by simple arithmetic across products; re-prescribe using the target product’s labeling/protocol and document the switch rationale
Renal dependence and accumulation propensity	Degree of renal clearance and accumulation risk differs with declining kidney function and prolonged courses	In CKD/AKI or fluctuating renal function, the same nominal regimen can behave differently over time	Require an explicit renal trajectory plan (dose/interval, reassessment timing, stop criteria); consider selective anti-Xa only when it will change action
Anti-Xa assay calibration dependence	Anti-Xa values depend on assay method/calibration; action-linked interpretation is required	Misinterpretation can create false precision and inappropriate dose changes—especially after switching	If anti-Xa is used, pre-specify an action plan and ensure the method is fit-for-purpose for the clinical question
Protamine “partial reversal” reality	Neutralization is incomplete for LMWH; reversal expectations should be realistic	Rescue planning differs from UFH-style “full reversal” assumptions in major bleeding/urgent procedures	In patients with high probability of urgent procedures/bleeding, incorporate reversal feasibility into selection; avoid late-course switches without a clear rescue pathway
Formulation/device + administration friction	Concentration, injection volume, and syringe presentation vary by product	Workflow frictions can increase dosing errors or missed doses	When switching for logistics, add a “dose-prep and administration check” (strength, rounding rule, education)

This table is designed to prevent “silent substitution.” when switching is unavoidable, it should trigger explicit re-checks of dose basis, renal trajectory, procedure timing, and (if used) action-linked anti-Xa interpretation rather than assuming class-level equivalence.

Row sources (expanded):

Row 1 (manufacturing/interchange): (Hogwood et al., 2023; Mulloy et al., 2016; Nenci, 2003; Merli and Groce, 2010; Fareed et al., 2004).

Row 2 (anti-Xa:anti-IIa, heterogeneity): (Hogwood et al., 2023; Mulloy et al., 2016; Merli and Groce, 2010).

Row 3 (units/labeling/non-interchangeable dosing): (U.S., food and drug administration, 2021; Merli and Groce, 2010).

Row 4 (renal impairment/accumulation and dosing guidance): (Hogwood et al., 2023; Mulloy et al., 2016; UK, kidney association Renal Association, 2023; UK, kidney association, 2025).

Row 5 (anti-Xa monitoring, calibration dependence, action-linked interpretation): (Jaspers et al., 2025; Thalappil et al., 2024; Verhamme et al., 2021).

Row 6 (protamine partial neutralization and clinical implications): (Hogwood et al., 2023; Mulloy et al., 2016; U.S., food and drug administration, 2021).

Row 7 (formulation/device and administration implications): (U.S., food and drug administration, 2021).

Abbreviations: LMWH, low-molecular-weight heparin; anti-Xa, anti–factor Xa activity; anti-IIa, anti–factor IIa (thrombin) activity; IU, international units; CKD, chronic kidney disease; AKI, acute kidney injury; UFH, unfractionated heparin.

### 4.2 PK/PD essentials: anti-Xa/anti-IIa activity, half-life, subcutaneous absorption, protein binding, distribution

Pharmacodynamically, LMWH activity is dominated by AT-dependent inhibition of factor Xa; inhibition of thrombin is conditional because it requires longer saccharide chains capable of bridging AT to thrombin (Hogwood et al., 2023; Mulloy et al., 2016). This chain-length dependence explains why lower mean molecular weight shifts activity toward anti-Xa relative to anti-IIa, and why a single anti-Xa value captures only part of the biologic anticoagulant effect (Hogwood et al., 2023; Mulloy et al., 2016; Amerali and Politou, 2022). LMWHs also promote tissue factor pathway inhibitor release and interact with cellular and inflammatory pathways, underscoring that pharmacodynamic effects extend beyond a single chromogenic readout (Hogwood et al., 2023; Mulloy et al., 2016).

Pharmacokinetically, subcutaneous administration provides high but context-sensitive bioavailability, with absorption shaped by local perfusion and tissue characteristics (Hogwood et al., 2023; Mulloy et al., 2016). Time-to-peak exposure depends on absorption kinetics and may be delayed when perfusion is impaired (Hogwood et al., 2023; Mulloy et al., 2016). Apparent distribution is predominantly intravascular with limited tissue diffusion; however, edema and critical illness may expand effective distribution and delay equilibration (Hogwood et al., 2023; Mulloy et al., 2016). Compared with unfractionated heparin, reduced nonspecific protein binding improves predictability, but binding and clearance remain influenced by acute-phase proteins and AT availability (Hogwood et al., 2023; Mulloy et al., 2016). The elimination half-life is longer than that of unfractionated heparin and varies by product and kidney function, enabling practical dosing regimens while making exposure sensitive to physiologic change (Hogwood et al., 2023; Mulloy et al., 2016; Merli and Groce, 2010). Collectively, this PK/PD variability supports a precision approach—selective dose tailoring and monitoring—when small exposure shifts are plausibly consequential for net clinical benefit (Hogwood et al., 2023; Mulloy et al., 2016).

### 4.3 Clearance and accumulation: kidney function, age, inflammation, and ICU physiology shaping exposure

LMWH clearance reflects both cellular uptake and renal elimination of smaller fragments; lower–molecular-weight products tend to be more renally dependent, making reduced kidney function—particularly in older adults—a primary driver of accumulation during repeated dosing (Hogwood et al., 2023; Mulloy et al., 2016; Merli and Groce, 2010). The same principle may operate in reverse in augmented renal clearance, in which high creatinine clearance during acute illness can lower exposure and contribute to under-anticoagulation despite standard dosing (Hogwood et al., 2023; Mulloy et al., 2016). Critical illness adds additional time-varying physiology: generalized edema and capillary leak can alter effective distribution, whereas vasopressors and peripheral hypoperfusion may reduce the reliability of subcutaneous absorption (Hogwood et al., 2023; Mulloy et al., 2016). Inflammatory states can also modify AT levels and heparin-binding proteins, plausibly shifting pharmacodynamics and clearance (Hogwood et al., 2023; Mulloy et al., 2016). In Coronavirus disease 2019 critical illness, pharmacokinetic observations with enoxaparin further illustrate that ICU states can produce exposure profiles that diverge from ambulatory expectations (Zuffe et al., 2021). In patients receiving intermittent venovenous hemofiltration, evidence supporting anti-Xa–guided dosing remains limited, and uncertainty persists regarding the extent to which extracorporeal circuits alter exposure (Xu et al., 2023).

### 4.4 Monitoring-relevant pharmacology: strengths and limits of anti-Xa as a surrogate endpoint

Anti-Xa monitoring is operationally attractive because assays are widely available, can be standardized within laboratories, and provide a drug-specific readout that is more direct than activated partial thromboplastin time for LMWH exposure assessment (Hogwood et al., 2023; Mulloy et al., 2016). However, its limitations are pharmacology-grounded and explain why “one-number targets” often lack portability across settings (Hogwood et al., 2023; Mulloy et al., 2016; Nenci, 2003). Anti-Xa results are highly dependent on sampling time relative to dosing (Hogwood et al., 2023; Mulloy et al., 2016; Nenci, 2003). Interpretation also varies with inter-assay and calibrator differences, including product-specific calibration, which constrains cross-laboratory comparability (Hogwood et al., 2023; Mulloy et al., 2016; Nenci, 2003). Mechanistically, anti-Xa reflects only AT-mediated Xa inhibition and incompletely represents anti-IIa activity and non-Xa pathways; therefore, it cannot serve as a global coagulation endpoint for LMWH effect (Hogwood et al., 2023; Mulloy et al., 2016; Nenci, 2003).

**FIGURE 1:**
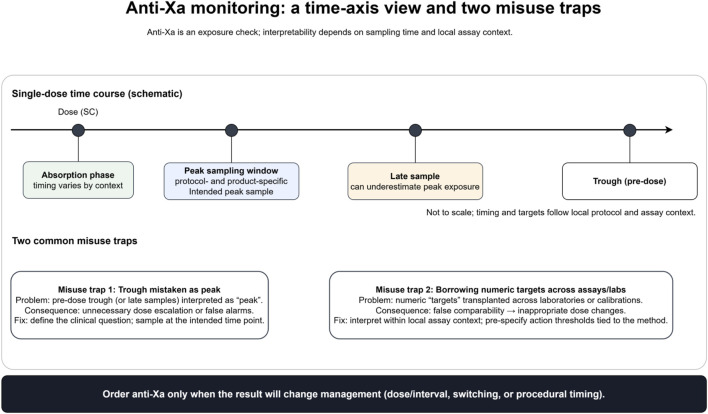
Anti-Xa monitoring: timing-dependent interpretation and common misuse traps. This schematic illustrates a single-dose time course and emphasizes that anti-Xa functions primarily as an exposure check rather than a surrogate efficacy endpoint; its interpretability depends on sampling time relative to dosing and the local assay context (Jaspers et al., 2025). Two frequent misuses drive “measurement without impact”: (i) mistiming (trough or late samples interpreted as peak exposure), which can prompt inappropriate dose changes, and (ii) threshold transport across laboratories or assay calibrations, which creates false comparability of numeric values (Jaspers et al., 2025; Thalappil et al., 2024). Anti-Xa testing should therefore be ordered selectively and interpreted only with a pre-specified action plan (Zuffe et al., 2021). Abbreviations: anti-Xa, anti–factor Xa activity; SC, subcutaneous; PK, pharmacokinetics; LMWH, low-molecular-weight heparin.

Figure 1 visualizes the dosing-to-sampling timeline and two high-frequency misuse traps: (i) mistiming (trough or late samples interpreted as peak) and (ii) threshold transport across assays or laboratories, both of which can convert monitoring into “measurement without impact” (Nenci, 2003; John et al., 2024). To operationalize the “non-portable threshold” principle, Supplementary Table S1 provides an audit-ready checklist for sampling and laboratory comparability (documentation of time since dose, calibrator and reagent strategy, units and reporting format, and defined “no-go” zones for cross-laboratory threshold transport) to complete before acting on numeric targets.

Consistent with these constraints, recent syntheses report inconsistent exposure–outcome correlations in many settings; anti-Xa is therefore best treated as an exposure check rather than a validated efficacy surrogate (Nenci, 2003; John et al., 2024). Global assays (e.g., thrombin generation) may serve as mechanistic adjuncts in research or selected evaluations, but they are not established tools for routine LMWH titration (Vermeiren et al., 2022). Accordingly, anti-Xa monitoring is most defensible when exposure uncertainty is high and results can plausibly change management (dose or interval adjustment, switching strategy, or procedural timing) (Nenci, 2003). Box 1 summarizes practical triggers for selective anti-Xa testing and pairs each trigger with an explicit action plan to avoid routine measurement without a management consequence (Nenci, 2003).

Box 1 **When anti-Xa monitoring is most defensible.**

Anti-Xa testing is most defensible only when exposure uncertainty is clinically consequential *and* results are expected to trigger a pre-specified management action. Sampling standards and action-linked interpretation are summarized in Section 6.2.

- Severe or fluctuating renal function: when accumulation or underexposure is plausible and would change bleeding/thrombosis risk and dose/interval decisions (Nenci, 2003).
- Extreme body size or altered physiology: when exposure uncertainty is clinically consequential (including pregnancy/postpartum *treatment* in selected high-stakes scenarios) (Nenci, 2003; Royal College of Obstetri cians and Gynaecologists RCOG, 2015a).
- Critical illness with non-stationary PK: when rapidly changing physiology (e.g., vasopressors, augmented renal clearance, CRRT/ECMO) makes exposure unpredictable, particularly during transitions of care (Jaspers et al., 2025; Zuffe et al., 2021; Xu et al., 2023).
- Unexpected clinical signal: when bleeding on prophylaxis, recurrence on treatment, or a discordant clinical–laboratory trajectory prompts a test that will inform dose adjustment or diagnostic reasoning (Nenci, 2003).
- Non-standard regimens with protocolized actions: when intensified prophylaxis or peri-procedural pathways are used and local protocols explicitly link anti-Xa results to defined actions (Nenci, 2003).

Abbreviations: anti-Xa, anti-factor Xa activity; CRRT, continuous renal replacement therapy; ECMO, extracorporeal membrane oxygenation; PK, pharmacokinetics.

To minimize false precision and reactive dose adjustments, anti-Xa testing should be ordered only when sampling can be standardized, and results will be acted upon through a pre-specified management plan. Supplementary Table S2 consolidates decision-relevant triggers, minimum sampling standards, and action-linked interpretation.

## 5 Clinical evidence map: prevention and treatment evidence landscape

**FIGURE 2:**
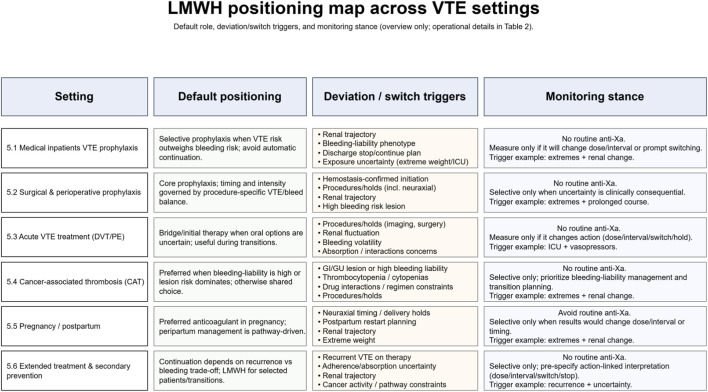
LMWH positioning map across VTE settings. A cross-setting overview of LMWH’s role in contemporary VTE care, organized by (i) default positioning, (ii) deviation/switch triggers, and (iii) an action-linked monitoring stance. Column shading is used to visually separate default positioning, trigger conditions, and monitoring statements; operational details are provided in Table 2 and the setting-specific subsections. Abbreviations: LMWH, low-molecular-weight heparin; VTE, venous thromboembolism; DVT, deep vein thrombosis; PE, pulmonary embolism; CAT, cancer-associated thrombosis; ICU, intensive care unit; anti-Xa, anti–factor Xa activity; GI, gastrointestinal; GU, genitourinary.

This section summarizes how LMWH is used across common VTE settings and focuses on practical questions: who should receive LMWH, at what dose intensity, for how long, when it should be held or restarted, and when another anticoagulant may be safer or easier to use (Khan et al., 2021; Stevens et al., 2021). To reduce repetition and preserve bedside usability, Figure 2 provides a one-page positioning map across settings (default positioning, deviation/switch triggers, and monitoring stance), and Table 2 translates this map into a cross-setting decision table designed for execution at the point of care. The subsections that follow provide (i) a brief take-home, (ii) one controversy thread, and (iii) one operational rule of thumb for each setting.

**TABLE 2:** LMWH decision table across VTE settings.

Setting	Default LMWH positioning	Exceptions/Switch triggers	Monitoring trigger (single phrase)
Medical inpatients (prophylaxis)	Standardized inpatient prophylaxis default for *selected* high-risk patients with revisitable bleeding risk	Mechanical-first/hold if bleeding risk dominates; stop at discharge unless an explicit extension plan exists	Only if actionable + standardized sampling
Surgery/peri-procedural (prophylaxis)	Bundle-compatible prophylaxis once an auditable “safe start” is enabled by procedure-family hemostasis criteria	Mechanical-first/hold (or UFH pathway) when bleeding or re-operation volatility dominates; extend only for selected high-risk operations	Rare; documentation/fidelity > assay
Acute VTE/PE (treatment)	Parenteral reliability when oral initiation is operationally fragile (absorption, interactions, perioperative timing) or physiology/renal trajectory is unstable	Switch to DOAC/VKA once stable and follow-up secured; use UFH when rapid titration/on–off is required (imminent procedures/extreme volatility)	Renal/extremes/ICU only if action-linked
Cancer-associated thrombosis (CAT; treatment)	LMWH-preferred when bleeding-liability management and interruptibility dominate (GI/GU phenotypes, luminal lesions, thrombocytopenia, interaction-heavy regimens, procedural cadence)	DOACs for selected lower-bleeding-risk phenotypes with disciplined interaction checks; UFH transiently around procedures/extreme volatility; reassess at regimen/platelet/procedure transitions	Selective; avoid number-chasing
Pregnancy/postpartum (prophylaxis + treatment)	Risk-structured prophylaxis-and-treatment pathway anchored in maternal–fetal safety and explicit peri-delivery execution (hold/restart rules)	Mechanical methods as complement/temporary substitute when bleeding risk is high; consider UFH near delivery when rapid on/off is required; postpartum switch only when feasible and follow-up secured	Selective; strict timing + action plan
Extended/secondary prevention	Selected constraint-heavy scenarios (oral unsuitable or transition windows); not routine long-term default outside cancer/pregnancy	Prefer oral long-term strategies when feasible; consider reduced-intensity (de-intensification) vs. stop at a defined reassessment checkpoint; reassess at procedures/absorption/interaction changes	No routine; reassessment plan > assay

This one-page matrix summarizes where LMWH, is most implementable across major VTE, settings by pairing a default positioning statement with the most common triggers to deviate (switch, hold, or use alternatives). “Exceptions/switch triggers” are intentionally phrased as operational cues rather than exhaustive criteria; granular phenotyping (e.g., renal trajectory, extremes of body weight, procedure cadence, bleeding phenotype, and oral-pathway fragility) should be specified in local pathways and in Supplementary Table S2 (monitoring triggers) and S4 (switch/alternatives). “Monitoring trigger” refers to anti-Xa activity testing and is listed only when (i) sampling time can be standardized and documented and (ii) a pre-specified action plan exists for results that are higher or lower than expected; otherwise, testing risks false precision and reactive dose changes. The “safe start” concept in surgery denotes initiation after adequate hemostasis by a service-level definition aligned with peri-procedural bleeding risk and re-operation probability. Postpartum “switch” implies transition only when follow-up is secured and patient preference and feasibility are aligned. The synthesis draws on contemporary international guidelines and cross-setting evidence maps (Stevens et al., 2021; Bistervels et al., 2022; Farge et al., 2022; Marcucci et al., 2022; Jaspers et al., 2025; Eck et al., 2022; National Institute for Health and Care Excellence, 2018).

Abbreviations: CAT, cancer-associated thrombosis; DOAC, direct oral anticoagulant; GI, gastrointestinal; GU, genitourinary; LMWH, low-molecular-weight heparin; PE, pulmonary embolism; UFH, unfractionated heparin; VKA, vitamin K antagonist; VTE, venous thromboembolism, anti-Xa, anti-factor Xa.

How to read this section. We organize the evidence map into six implementation-relevant settings—medical inpatients (prophylaxis), surgery/periprocedural prophylaxis, acute DVT/PE treatment, cancer-associated thrombosis, pregnancy/postpartum, and extended/secondary prevention—because LMWH’s real-world value is most sensitive to selection boundaries and transition execution rather than to any single “best agent” claim. Figure 2 is intended as the visual navigation for this section; Table 2 provides the one-page cross-setting skeleton. An expanded scenario-by-decision matrix is provided in Supplementary Table S3. Monitoring is addressed in two layers to avoid false precision: Box 1 lists triggers for selective anti-Xa testing, while Section 6.2 summarizes sampling standards and action-linked interpretation. Auditable switching and alternative prompts are consolidated in Supplementary Table S4.

As shown in Table 2, uncertainty concentrates less in whether LMWH “works” and more in patient selection, safe-start definitions, transitions, and low-burden monitoring that is explicitly tied to management actions.

### 5.1 Medical inpatients VTE prophylaxis: from “use or not” to “how far to go”

In medical inpatients, LMWH is best positioned as a standardized inpatient prophylaxis default for selected high-risk patients; uncertainty and practice variation concentrate in “how far to go,” particularly in selection boundaries and decisions about extension beyond discharge (Eck et al., 2022; National Institute for Health and Care Excellence, 2018). Network meta-analytic evidence indicates that pharmacologic prophylaxis reduces VTE in selected acutely ill medical inpatients but increases major bleeding, underscoring that expanded coverage should be deliberate rather than automatic (Eck et al., 2022).

RAMs can support selectivity, but recent comparative evidence shows limited discrimination when RAMs are used as stand-alone gatekeepers (Häfliger et al., 2024). In practice, RAMs perform more reliably as pathway prompts—paired with explicit bleeding-contraindication checks, periodic reassessment as physiology evolves, and auditing of both omissions and inappropriate continuation (National Institute for Health and Care Excellence, 2018).

Extended post-discharge prophylaxis is the most contested “how far” decision because thrombotic risk may persist after discharge while bleeding risk, adherence, and monitoring capacity shift outside the hospital (Khan et al., 2021). The MICHELLE trial showed that selected high-risk patients discharged after COVID-19 hospitalization could benefit from extended thromboprophylaxis with an oral factor Xa inhibitor. However, because MICHELLE enrolled a COVID-19 cohort, its generalizability to broader acutely ill medical inpatients remains uncertain; therefore, it should not be interpreted as establishing a universal post-discharge extension rule (Ramacciotti et al., 2022). Trial evidence in enriched inpatient phenotypes further reinforces that observed benefit depends primarily on *who is selected*, not on the agent label alone (Mottier et al., 2023).

Practical take-home. In medical inpatients, LMWH is best positioned as a standardized inpatient prophylaxis default for selected high-risk patients with acceptable, reassessable bleeding risk; the main implementation challenge is defining selection and post-discharge extension boundaries.

Controversy. Practice variation concentrates in the limited bedside discrimination of RAMs when used as stand-alone gatekeepers and in identifying discharge phenotypes in which residual thrombotic risk remains high enough to justify extension under real-world bleeding, adherence, and monitoring constraints.

Rule of thumb. Treat “medical prophylaxis” as a pathway decision (contraindication-aware and reassessed as physiology evolves) rather than a one-time RAM score. Stop LMWH at discharge unless an explicitly documented extension plan exists. Reserve therapeutic-intensity LMWH for confirmed incident VTE and avoid conflating prophylaxis with treatment.

### 5.2 Surgical and perioperative prophylaxis: timing, intensity, and bleeding liability

In surgery, LMWH remains a core prophylaxis option, but net benefit is governed by procedure-specific bleeding liability and by how teams operationalize and document a “safe start” (Stevens et al., 2021; Samama et al., 2024). The evidence map supports stratified pathways (procedure risk × patient risk), often layered with mechanical methods when VTE risk is extreme (National Institute for Health and Care Excellence, 2018; Samama et al., 2024).

Risk trajectories differ across operations: major orthopedic and oncologic procedures often sustain postoperative VTE risk longer than many general-surgery populations, while bleeding consequences are immediate and procedure-dependent (National Institute for Health and Care Excellence, 2018; Samama et al., 2024). Across non-cardiac surgery, network meta-analytic evidence suggests that anticoagulant prophylaxis reduces VTE but can increase major bleeding; therefore, “more intensive” or “longer” is not inherently “better” (Stevens et al., 2021). The most actionable implementation step is aligning institutional defaults (including duration prompts) with procedure families rather than relying on *ad hoc* prescriber variation (National Institute for Health and Care Excellence, 2018). Mechanical prophylaxis targets stasis and offers an option when bleeding risk is prohibitive, while combined mechanical-plus-pharmacologic approaches are used when VTE risk is high and bleeding risk is acceptable (National Institute for Health and Care Excellence, 2018; Samama et al., 2024).

#### 5.2.1 Controversy 2: early postoperative initiation versus hemostasis-confirmed initiation

The operational question is not whether teams should “start early” in the abstract, but what qualifies as adequate hemostasis for a given procedure family and bleeding-risk phenotype. European perioperative guidance and National Institute for Health and Care Excellence frame initiation as contingent on hemostasis and bleeding-risk phenotype rather than a fixed clock time (National Institute for Health and Care Excellence, 2018; Samama et al., 2024). This approach implies an auditable documentation requirement: locally defined criteria for “hemostasis adequate,” a named decision-maker (with escalation pathways), and reliable reassessment when clinical status evolves. Without shared criteria and documentation, “early start” becomes aspirational rather than auditable, and real-world safety can drift from the evidence base (Samama et al., 2024).

**FIGURE 3:**
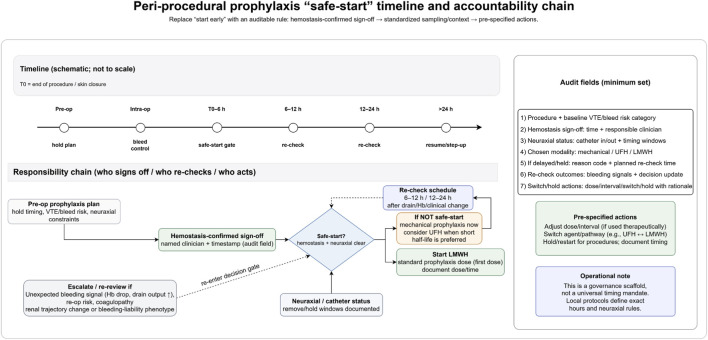
Peri-procedural prophylaxis “safe-start” timeline and accountability chain. The figure replaces nonspecific “start early” recommendations with an auditable rule for initiating LMWH prophylaxis after surgery. A hemostasis-confirmed sign-off (named clinician and timestamp) and documented neuraxial or catheter status form the decision gate (“safe-start”). If criteria are met, standard prophylactic LMWH may be initiated with dose and timing recorded. If not, mechanical prophylaxis is used and UFH may be considered when rapid reversibility is preferred. Unexpected bleeding signals or changes in clinical risk trigger escalation and re-entry into the decision gate rather than automatic action. Re-checks are scheduled at predefined intervals (e.g., 6–12 h or 12–24 h) or after clinical changes. The timeline is schematic and not to scale; exact timing and neuraxial constraints are defined by local protocols. Abbreviations: LMWH, low-molecular-weight heparin; UFH, unfractionated heparin; VTE, venous thromboembolism.

To operationalize this distinction, Figure 3 translates “early initiation” into an auditable periprocedural safe-start workflow anchored on hemostasis-confirmed sign-off, accountable decision-making, and predefined reassessment and escalation.

Practical translation. Convert “start early” into an auditable “safe start” rule: initiation should be contingent on locally defined, procedure-family–specific hemostasis criteria, an accountable decision-maker (with escalation), and reassessment when bleeding risk or clinical status changes. Mechanical prophylaxis should dominate when bleeding risk outweighs thrombotic risk, and longer courses should be reserved for explicitly high-risk operations.

Guardrail. This section addresses prophylaxis; therapeutic-intensity anticoagulation should be activated only for confirmed incident postoperative VTE and should not be embedded in routine prophylaxis decisions.

See Table 2; Supplementary Tables S2, S4 for the cross-setting decision table, monitoring triggers, and switch/alternative prompts.

### 5.3 Acute VTE treatment: LMWH’s role in initial and transition therapy

In acute DVT/PE, LMWH is most structurally valuable as immediately effective parenteral therapy that supports stabilization and controlled transitions when oral therapy is constrained by physiology, drug–drug interactions, or periprocedural timing (Kahn and de Wit, 2022; Stevens et al., 2021). DOACs simplify many pathways, but their advantage narrows when absorption, kidney function, or bleeding volatility is uncertain (Freund et al., 2022; Maughan et al., 2025).

Outpatient treatment and early discharge are feasible for selected low-risk PE patients when diagnostic certainty, stability, and follow-up capacity are in place (Kahn and de Wit, 2022; Freund et al., 2022). In such pathways, DOACs often reduce operational complexity; however, LMWH remains useful when same-day oral initiation is delayed, when nausea or periprocedural restrictions threaten absorption, or when a short “bridge” is preferred while comorbid risk is clarified (Freund et al., 2022).

When LMWH is chosen over a DOAC or vitamin K antagonist, the decision logic is typically exposure certainty and interruptibility: unstable kidney function, unreliable gastrointestinal absorption, high-risk drug–drug interactions, recent surgery, or rapidly changing physiology (including critical illness) favor parenteral therapy (Kahn and de Wit, 2022; Freund et al., 2022; Stevens et al., 2021). Contemporary syntheses emphasize phenotype- and setting-aligned selection, with structured pathways to reduce avoidable variation without enforcing a single default agent (Maughan et al., 2025).

LMWH can serve as a control-oriented parenteral option, where “control” refers to exposure predictability and practical interruptibility across transitions rather than superior efficacy. Unfractionated heparin (UFH) is preferred when near-continuous titration or very rapid on–off control is required (e.g., imminent procedures or extreme bleeding volatility). LMWH is often preferable when oral strategies are operationally fragile (absorption uncertainty, interaction burden, periprocedural timing constraints, or unstable renal trajectory) and a portable strategy with lower monitoring burden is needed (Stevens et al., 2021). From an implementation perspective, parenteral selection is therefore a physiology-and-workflow decision that can be audited through documented transition checkpoints and unplanned switching frequency, not merely clinician preference (Kahn and de Wit, 2022).

Practical take-home. In acute VTE/PE, LMWH is most useful as immediately effective parenteral therapy when exposure certainty and interruptibility are decisive—particularly when oral initiation is operationally fragile or kidney/physiologic trajectories are unstable—while UFH should be preferred when near-continuous titration or very rapid interruption is required.

Controversy. The key uncertainty is not whether anticoagulation is indicated, but when and how to transition to an oral strategy and embed phenotype-based choices into consistent, auditable pathways without enforcing a single default agent.

See Table 2; Supplementary Tables S2, S4 for the cross-setting decision table, monitoring triggers, and switch/alternative prompts.

### 5.4 Cancer-associated thrombosis (CAT): where LMWH remains preferred and where DOAC evidence applies

In cancer-associated thrombosis (CAT), LMWH remains a first-line option for higher-bleeding-risk phenotypes and for patients with complex interaction profiles, while DOAC trials delineate an evidence boundary that depends on tumor site, mucosal bleeding risk, and treatment context (Farge et al., 2022; Key et al., 2023). Net benefit is best framed as recurrence prevention versus site-specific bleeding liability in the individual patient, rather than as a single global ranking across all cancers (Farge et al., 2022).

Randomized trials comparing DOACs with LMWH (including Hokusai VTE Cancer trial, SELECT-D trial, and CARAVAGGIO trial) support that oral factor Xa inhibition can be effective for preventing recurrent VTE across broad cancer populations; however, bleeding patterns differ and appear concentrated in specific tumor and lesion contexts (Agnelli et al., 2020; Raskob et al., 2018; Young et al., 2018). CARAVAGGIO trial and subsequent bleeding analyses further reinforce that “cancer” is not a uniform bleeding phenotype and that tumor location, mucosal involvement, recent procedures, and concurrent therapies are central to anticoagulant selection (Merli and Groce, 2010). These trial-defined boundaries map directly onto guideline language that frames DOAC use as selective rather than universal in CAT (Farge et al., 2022; Key et al., 2023).

#### 5.4.1 Controversy 3: DOAC-first convenience versus LMWH-preferred strategies for bleeding-liability management

This controversy often reflects different default priorities—maximizing convenience and persistence versus minimizing bleeding uncertainty in higher-risk phenotypes (Farge et al., 2022; Key et al., 2023). A clinically executable approach begins with explicit phenotype definition (e.g., gastrointestinal/genitourinary tumors, active luminal lesions, thrombocytopenia, interacting systemic therapy, recent invasive procedures), then matches anticoagulant selection to the required interruptibility and tolerance for bleeding uncertainty (Farge et al., 2022; Key et al., 2023). In patients with CAT and thrombocytopenia, platelet count should be incorporated into the anticoagulant plan. Full-dose LMWH is generally considered acceptable when platelet counts exceed 50,000/μL and there is no active bleeding; lower platelet counts require individualized dose reduction, temporary interruption, platelet support, or alternative strategies according to thrombotic risk, bleeding risk, and institutional protocols (Lyman et al., 2021; Patell et al., 2022). Implementation is frequently decisive: injection burden and access barriers can erode LMWH persistence, whereas DOAC simplification may improve feasibility but requires disciplined interaction checks and bleeding surveillance, particularly across treatment transitions (Key et al., 2023). Real-world comparative evidence showing frequent switching between LMWH and DOAC strategies highlights that trial efficacy and system effectiveness can diverge when adherence, access, and transitions dominate outcomes (Kang et al., 2024).

Practical take-home. In established CAT, anticoagulant selection should be phenotype-first: LMWH is preferred when bleeding-liability management, interruptibility, absorption uncertainty, or interaction burden makes a DOAC-first strategy operationally brittle; DOACs are reasonable in selected lower-bleeding-risk phenotypes when interaction checks, and bleeding surveillance can be delivered reliably.

Controversy. The central uncertainty is not abstract class efficacy, but how services operationalize gastrointestinal/genitourinary bleeding-risk phenotypes and whether frequent real-world switching improves or undermines net clinical benefit outside trial settings.

See Table 2; Supplementary Tables S2, S4 for the cross-setting decision table, monitoring triggers, and switch/alternative prompts.

### 5.5 Pregnancy/postpartum and other special life stages: LMWH in limited-substitutability settings

**FIGURE 4:**
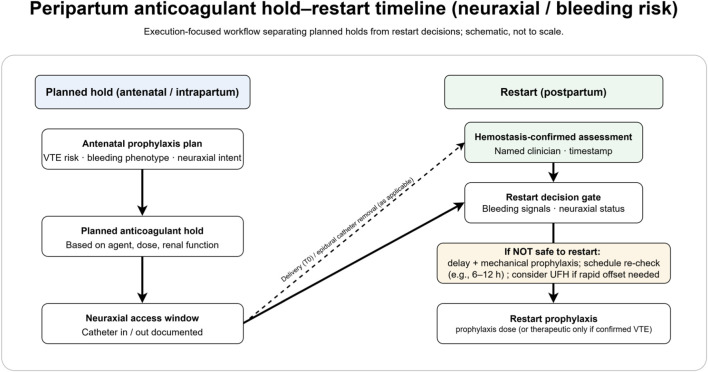
Peripartum anticoagulant hold–restart workflow (neuraxial/bleeding risk). Schematic, not to scale. The workflow separates antenatal/intrapartum planned holds (agent-, dose-, and renal function–dependent) from postpartum restart decisions anchored to hemostasis confirmation and neuraxial status, with documentation of the responsible clinician and timestamp. When restart is not safe, anticoagulation is deferred with mechanical prophylaxis and scheduled reassessment according to local protocols. Typical minimum pre-neuraxial hold intervals are ≥12 h for prophylactic-dose LMWH and ≥24 h for therapeutic-dose LMWH; final timing should follow local anesthesia protocols and account for renal function and bleeding risk. Abbreviations: LMWH, low-molecular-weight heparin; UFH, unfractionated heparin; VTE, venous thromboembolism.

In pregnancy and the postpartum period, LMWH remains a preferred anticoagulant because it has established maternal–fetal safety experience and because DOACs are generally avoided in this setting (Royal College of Obstetricians and Gynaecologists RCOG, 2015b; American Society of Hematology, 2025; European Society of Cardiology (ESC), 2025). However, LMWH management in pregnancy is not limited to agent selection. Dose intensity and timing may need reassessment as body weight, renal clearance, bleeding risk, delivery plans, and neuraxial anesthesia needs change across gestation and after delivery (Royal College of Obstetricians and Gynaecologists RCOG, 2015b; American Society of Hematology, 2025; European Society of Cardiology (ESC), 2025). A practical peripartum plan should specify the intended antepartum dose, the last planned antenatal dose, what to do if spontaneous labor or vaginal bleeding occurs, the minimum interval before neuraxial anesthesia, and the criteria for postpartum restart after hemostasis assessment. Figure 4 separates planned holding decisions before delivery from restart decisions after delivery and emphasizes that postpartum restart should be based on hemostasis, neuraxial catheter status, dose intensity, and ongoing VTE risk rather than on a fixed clock time alone. For neuraxial procedures, prophylactic-dose LMWH should generally be withheld for at least 12 h and therapeutic-dose LMWH for at least 24 h before neuraxial anesthesia or catheter placement. Final timing should be confirmed against renal function, dose intensity, catheter status, bleeding risk, and institutional anesthesia protocols (Royal College of Obstetricians and Gynaecologists RCOG, 2015b; American Society of Hematology, 2025; European Society of Cardiology (ESC), 2025).

Highlow trial demonstrates that prophylaxis intensity (intermediate-dose versus low-dose LMWH) is an evidence-tested decision in women with prior VTE, anchoring disagreements about whether higher intensity improves prevention sufficiently to justify added bleeding risk or burden in selected phenotypes (Bistervels et al., 2022). Royal College of Obstetricians and Gynaecologists, American Society of Hematology, and European Society of Cardiology guidance operationalize this through risk-stratified pathways and peridelivery execution rules, emphasizing planned holding for neuraxial anesthesia and bleeding risk and structured restart once hemostasis is acceptable (Royal College of Obstetricians and Gynaecologists RCOG, 2015b; American Society of Hematology, 2025; European Society of Cardiology (ESC), 2025). Postpartum prophylaxis remains an implementation stress test: risk is front-loaded but heterogeneous, so intensity and duration decisions are best treated as stratified (and revisited) rather than “one duration fits all” (Royal College of Obstetricians and Gynaecologists RCOG, 2015a).

Practical take-home. In pregnancy and postpartum, LMWH should be managed as a risk-structured prophylaxis-and-treatment pathway anchored in maternal–fetal safety and explicit peridelivery execution (planned holds for neuraxial anesthesia and bleeding risk, followed by structured restart once hemostasis is acceptable), rather than as convenience-driven agent selection.

Controversy. The key uncertainty is how to calibrate prophylaxis intensity and postpartum duration for intermediate-risk phenotypes, and whether adjustment or monitoring strategies can improve net benefit without adding undue burden or false precision.

Rule of thumb. Pre-specify peripartum coordination (who decides hold/restart, escalation pathways, and reassessment triggers) and manage postpartum prophylaxis as stratified-and-revisited rather than “one duration fits all.” Use UFH selectively near delivery only when rapid on–off control is operationally required.

See Table 2; Supplementary Tables S2 and S4 for the cross-setting decision table, monitoring triggers, and switch/alternative prompts.

### 5.6 Extended treatment and secondary prevention: duration, de-intensification, and stopping decisions

After the initial treatment phase, LMWH is not the routine long-term strategy for most non-cancer, non-pregnancy patients, but it remains a practical option when oral therapy is unsuitable and during transition windows in which procedures, absorption, or interaction profiles change (Stevens et al., 2021). Secondary prevention decisions hinge on duration and intensity—recurrence prevention versus accumulating bleeding risk—under substantial residual uncertainty (Kimpton and Wang, 2025). RENOVE study supports reduced-intensity anticoagulation as a de-intensification option after unprovoked VTE, reinforcing that “continue versus stop” is often better framed as “full-dose versus reduced-dose versus stop” under individualized risk trade-offs (Kimpton and Wang, 2025; Couturaud et al., 2025).

Practical take-home. After the initial treatment phase, extended management is primarily a duration-and-intensity decision. LMWH is best reserved for selected patients in whom oral strategies are unsuitable or during short transition windows when oral therapy becomes operationally fragile.

Controversy. The dominant uncertainty is operational: how to standardize stopping decisions and communicate evolving recurrence and bleeding risks as patient priorities, comorbidities, and feasibility change over time.

Rule of thumb. Reframe “continue versus stop” as a three-option pathway—full-intensity versus reduced-intensity versus stop—reassessed at a defined checkpoint after the initial treatment window. Favor de-intensification when net benefit likely persists but bleeding risk or burden accumulates, and reduced-intensity oral strategies are feasible.

See Table 2; Supplementary Tables S2 and S4 for the cross-setting decision table, monitoring triggers, and switch/alternative prompts.

## 6 Controversies and implementation: how schools of thought translate to bedside practice

### 6.1 Fixed dosing vs. weight-/risk-stratified dosing: reproducibility vs. precision

Two competing approaches dominate LMWH dosing strategy: fixed prophylaxis defaults that prioritize reproducibility versus patient-level weight and risk stratification intended to improve exposure precision (Hogwood et al., 2023). Fixed dosing emphasizes deliverability and auditability; when missed doses, delayed initiation, and inconsistent execution are the dominant failure modes, a robust default may improve net clinical benefit even if individualization is imperfect (National Institute for Health and Care Excellence, 2018; Vincent et al., 2022). Stratification is most defensible when baseline thrombotic risk is high or exposure uncertainty is substantial (e.g., extremes of body weight, renal dysfunction, or rapidly changing critical illness), because underexposure can undermine preventive or therapeutic intent (Hogwood et al., 2023; Vincent et al., 2022). However, additional complexity introduces predictable failure modes—risk misclassification, dosing arithmetic errors, and inconsistent implementation—and any theoretical gain must exceed harms from operational fragility and bleeding from overtitration (National Institute for Health and Care Excellence, 2018; Vincent et al., 2022). When uncertainty is high and systems are not designed to execute complex algorithms reliably, execution often matters more than incremental dose refinement (Vincent et al., 2022). Rule of thumb: start with a guideline-concordant default and escalate to a written stratification algorithm only when thrombotic stakes are high *and* the team can implement it consistently (including prescribing, administration, and audit loops) (National Institute for Health and Care Excellence, 2018; Vincent et al., 2022).

### 6.2 Anti-Xa monitoring: routine-monitoring camp vs. selective-monitoring camp

Two schools compete: routine anti-Xa monitoring to “control” LMWH therapy versus selective monitoring used only when exposure uncertainty is clinically consequential. Critically, the intended endpoint should be declared upfront—preventing bleeding from accumulation, avoiding recurrence from underexposure, or providing reassurance—because these aims imply different action thresholds and different tolerance for uncertainty (Nenci, 2003; Saunders et al., 2024). Systematic reviews and outcome studies show mixed or setting-dependent benefit from anti-Xa–guided dose adjustment, and routine testing can devolve into “measurement without impact,” particularly when sampling is non-standardized and trough-like samples are interpreted as “low” or peak-timed samples as “high” (Nenci, 2003; Saunders et al., 2024). Inter-laboratory assay and calibrator variability, coupled with the surrogate nature of anti-Xa, limits threshold portability and weakens assumptions of a stable exposure–outcome relationship across prophylaxis versus treatment or across assays (Nenci, 2003; Thalappil et al., 2024). Rule of thumb: use anti-Xa selectively with a pre-specified clinical question, standardized sampling time (with documented dose-to-draw interval), and an explicit action plan; avoid reactive dose changes based on isolated values that are timing-discordant or clinically incongruent (Nenci, 2003; Thalappil et al., 2024). To make this selective-monitoring approach more clinically usable, Box 2 provides common scenarios in which anti-Xa testing may be considered and links each scenario to a potential management action.

Box 2 **Practical examples of selective anti-Xa monitoring.**

The following examples illustrate how selective anti-Xa monitoring can be linked to a management decision; they should be interpreted together with standardized sampling time, LMWH product, renal function, assay method, and local protocols (Nenci, 2003; Thalappil et al., 2024; Saunders et al., 2024).

- Renal impairment or rapidly changing renal function. In a patient receiving therapeutic LMWH with severe or worsening renal impairment, a correctly timed anti-Xa level may support dose reduction, interval extension, or switching to UFH if accumulation is suspected (Nenci, 2003; UK Kidney Association Renal Association, 2023; UK Kidney Association, 2025).
- Extreme body weight. In severe obesity or very low body weight, anti-Xa testing may be considered when standard weight-based or fixed dosing raises concern for underexposure or overexposure, provided that the sampling time and local assay interpretation are clear (Nenci, 2003; Appay et al., 2025).
- Pregnancy with high thrombotic risk. In selected pregnant or postpartum patients with recurrent VTE risk, major weight change, renal-function change, or recurrent thrombosis despite treatment, anti-Xa testing may help determine whether dose adjustment is reasonable (Nenci, 2003; Royal College of Obstetricians and Gynaecologists RCOG, 2015a; Royal College of Obstetricians and Gynaecologists RCOG, 2015b).
- Unexpected bleeding or recurrent thrombosis. Anti-Xa testing may be useful when bleeding occurs on LMWH or thrombosis recurs despite apparent adherence. In these cases, the result should be interpreted together with last-dose timing, renal function, body weight, LMWH product, adherence, interacting drugs, and the possibility of disease progression or HIT (Nenci, 2003; Warkentin and Greinacher, 2016).
- Mistimed samples. A trough, late sample, or undocumented sample should not be used alone to increase or decrease the LMWH dose (Nenci, 2003; Thalappil et al., 2024).

### 6.3 Renal impairment and accumulation: dose reduction, switching, or alternative anticoagulation?

Two strategies are commonly advocated in renal impairment: dose-reducing LMWH to mitigate accumulation versus switching to an alternative anticoagulant strategy (often unfractionated heparin [UFH]) when accumulation risk predominates (UK Kidney Association Renal Association, 2023; UK Kidney Association, 2025). A recurrent pitfall is treating kidney function as static. As renal function declines—or fluctuates during admission—LMWH exposure can rise and bleeding risk can increase; therefore, renal function should be managed as a trajectory rather than as a single baseline estimate (UK Kidney Association Renal Association, 2023; U.S. Food and Drug Administration, 2021). Rule of thumb: when renal dysfunction is advanced or rapidly changing, favor an easily titratable, rapidly reversible approach (e.g., UFH) and minimize prolonged LMWH exposure. Otherwise, follow renal-dose guidance, avoid unnecessary interruptions during transitions, and reassess frequently as renal trajectory evolves (UK Kidney Association Renal Association, 2023; U.S. Food and Drug Administration, 2021; Vincent et al., 2022; UK Kidney Association, 2025).

### 6.4 Extreme body weight (obesity/low weight) and uncertainty in subcutaneous absorption

Two approaches compete at extremes of habitus: weight-based therapeutic dosing to avoid underexposure in obesity versus capped or de-intensified dosing intended to reduce bleeding risk and operational error. Across obesity and low body weight, systematic reviews describe heterogeneous regimens and limited certainty regarding optimal dosing strategy (Appay et al., 2025; Agnelli et al., 2020). The central implementation risk is assuming linear dose–exposure scaling at the extremes: subcutaneous delivery, distribution, and “effective exposure per milligram” can be context-sensitive, making both underexposure (in obesity) and overexposure with bleeding vulnerability (in low body weight) plausible in practice (Hogwood et al., 2023; Appay et al., 2025; Agnelli et al., 2020). Rule of thumb: begin with a transparent, weight-based plan and avoid *ad hoc* caps. Reserve anti-Xa as a selective problem-solving adjunct only when results will change management and sampling/assay conditions can be standardized, because timing dependence and assay variability can otherwise misdirect dose decisions (Nenci, 2003; Appay et al., 2025; Thalappil et al., 2024).

### 6.5 Peri-procedure “bridging”: who truly needs it, and what is inertia care?

Two schools compete: routine peri-procedure bridging to prevent thrombosis versus minimalist bridging to reduce bleeding, complexity, and delays in achieving postoperative hemostasis (Hogwood et al., 2023; Douketis et al., 2022). CHEST guidance frames bridging as an exception for narrowly defined high-risk situations rather than a default, because procedure-related bleeding liability and postoperative hemostasis uncertainty often outweigh theoretical thromboembolic protection (Douketis et al., 2022; Lyons et al., 2024). In PERIOP2 trial, routine postoperative LMWH bridging in warfarin-treated patients did not demonstrate a clear reduction in thromboembolism and increased bleeding-related burden, supporting restraint as the default posture (Kovacs et al., 2021). “Inertia care” refers to bridging that persists because it is embedded in order sets or inherited plans, rather than because the current patient’s thrombosis-versus-bleeding balance supports it (Lyons et al., 2024). Rule of thumb: bridge only when interruption confers clearly high thromboembolic risk *and* postoperative anticoagulation can be restarted safely on a planned timetable; otherwise, do not bridge (Douketis et al., 2022; Lyons et al., 2024).

## 7 Safety and risk management: Adverse effects, reversal, and governable risk

### 7.1 Bleeding: risk stratification, reversible drivers, and management pathways

Bleeding is the predictable trade-off of anticoagulation; therefore, LMWH safety begins with pre-dose risk stratification and an explicit plan for reassessment as physiology, co-medications, and procedural timing evolve (Stevens et al., 2021; Lyons et al., 2024). Many apparently “unexpected” bleeding events have governable drivers, including renal impairment with drug accumulation, concomitant antiplatelet agents or nonsteroidal anti-inflammatory drugs, periprocedural timing errors, and dosing or product-switch mistakes (Hogwood et al., 2023; Fareed et al., 2004; Lyons et al., 2024). Bedside management should be protocolized: hold LMWH, pursue local hemostatic control, assess hemoglobin, platelet count, and kidney function, obtain imaging promptly when clinically indicated, and provide transfusion support when necessary (Stevens et al., 2021; U.S. Food and Drug Administration, 2021; Lyons et al., 2024). Medication reconciliation should discontinue nonessential antiplatelets and nonsteroidal anti-inflammatory drugs and confirm last-dose timing, because preventable accumulation and timing errors are common contributors (Hogwood et al., 2023; U.S. Food and Drug Administration, 2021). Clinicians should also reassess the anticoagulation indication and short-term thrombotic risk, because premature discontinuation can be harmful in patients with recent VTE (Stevens et al., 2021; Nenci, 2003). Once bleeding is controlled, restart decisions should be anchored to procedural hemostasis and VTE risk, with a documented plan for timing and follow-up (Stevens et al., 2021; Lyons et al., 2024). Early escalation to hematology and procedural teams is appropriate for uncontrolled bleeding or complex restart decisions, and cases should be fed back into pathway governance to support audit and prevention (Stevens et al., 2021; Lyons et al., 2024).

### 7.2 Reversal and emergencies: practical limits of protamine and bedside strategies

Major bleeding emergencies highlight the practical limits of “reversal” for LMWH. Protamine can neutralize a portion of anticoagulant activity but does not reliably restore baseline hemostasis, particularly once distribution and clearance have progressed (Hogwood et al., 2023; U.S. Food and Drug Administration, 2021). Accordingly, bedside strategy should be organized around time since last dose, immediate discontinuation, local or procedural bleeding control, and supportive care (volume resuscitation, transfusion support, and rapid escalation to procedural teams), rather than an expectation of complete pharmacologic reversal (Hogwood et al., 2023; U.S. Food and Drug Administration, 2021). When LMWH is being used for acute VTE, re-initiation planning should be explicit once hemostasis is secured, because prolonged interruption re-exposes patients to thromboembolic risk (U.S. Food and Drug Administration, 2021).

### 7.3 Heparin-induced thrombocytopenia (HIT), osteoporosis, skin necrosis, and injection-related problems: recognition and response

Beyond bleeding, two governance-relevant safety domains warrant active surveillance: heparin-induced thrombocytopenia (HIT) and longer-horizon toxicities associated with prolonged heparin exposure (Hogwood et al., 2023; Warkentin and Greinacher, 2016). HIT should be treated as time-critical when platelet counts fall within a compatible exposure window or new thrombosis occurs, because the syndrome is prothrombotic rather than “isolated thrombocytopenia” (Warkentin and Greinacher, 2016; Cuker et al., 2018). Immediate management is to stop all heparin products (including LMWH), avoid heparin flushes, and initiate a non-heparin anticoagulant when ongoing anticoagulation is indicated (Warkentin and Greinacher, 2016; Cuker et al., 2018). Platelet transfusion is generally avoided unless there is active bleeding or an urgent procedure, because transfusion may exacerbate a prothrombotic state and complicate management (Cuker et al., 2018). With prolonged use, osteoporosis is a recognized concern, reinforcing the need to reassess duration and transition to alternatives when feasible (Hogwood et al., 2023). Rare skin necrosis or severe injection-site reactions warrant prompt discontinuation, evaluation for HIT or hypersensitivity, and re-planning of anticoagulation supported by patient-centered counseling (U.S. Food and Drug Administration, 2021; Warkentin and Greinacher, 2016). For recurrent bruising or nodules, site rotation, technique review, and documentation of intolerance can reduce preventable discontinuation and support appropriate switching decisions (U.S. Food and Drug Administration, 2021).

### 7.4 Patient experience and adherence: injection burden, education, and follow-up

LMWH’s injection burden is a safety and effectiveness issue because missed or mistimed doses can convert an otherwise manageable plan into ungoverned exposure variability (Khan et al., 2021; Stevens et al., 2021). Education should address injection technique, site rotation, and clear thresholds for what bruising is expected versus when to report pain, swelling, or bleeding symptoms (Stevens et al., 2021). Structured follow-up touchpoints—such as an early phone check, refill verification, and reassessment at transitions of care—support adherence and facilitate shared decision-making to switch to oral options when clinically appropriate and feasible for the patient (Khan et al., 2021; Stevens et al., 2021).

## 8 Research gaps and future directions: what is truly missing is explainable and implementable evidence

### 8.1 Key evidence gaps by population and context

The field does not require additional reviews; it requires exposure–response clarity, actionable stratification for net clinical benefit (thrombosis versus bleeding), and study designs that generate bedside rules and health-system defaults (Stevens et al., 2021). The most consequential gaps cluster in phenotypes for which LMWH is chosen to improve predictability, yet exposure variability and competing risks are greatest (Stevens et al., 2021).

- Renal dysfunction (including advanced chronic kidney disease).
- Uncertain: how renal trajectory (rather than a single baseline estimate) maps to bleeding and recurrent VTE risk, and when dose reduction should transition to switching strategies beyond anti-Xa values (Nenci, 2003; UK Kidney Association, 2025).
- Bias: anti-Xa is used as a safety anchor and “renal thresholds” become local convention (Nenci, 2003; UK Kidney Association, 2025).
- Decisive evidence: time-updated exposure–outcome analyses and strategy comparisons that yield explicit dose/hold/switch triggers linked to patient outcomes (UK Kidney Association, 2025; Hernán et al., 2022).
- Extreme body weight and subcutaneous absorption uncertainty.
- Uncertain: the predictability of subcutaneous delivery and whether caps or fixed doses create underexposure or excess bleeding risk at extremes (Stevens et al., 2021).
- Bias: default linearity (dose proportional to weight) or one-size rules because absorption is unmeasured and anti-Xa is an incomplete surrogate (Nenci, 2003).
- Decisive evidence: prospective testing of rule sets using sparse sampling and clinical endpoints, not merely “target” attainment (Nenci, 2003; Hernán et al., 2022).
- ICU and inflammation-driven physiology.
- Uncertain: pharmacokinetic instability with augmented renal clearance versus evolving organ dysfunction, and the cumulative impact of procedure-related holds that fragment intended exposure (Stevens et al., 2021).
- Bias: simplifying decisions and using monitoring for reassurance despite assay limitations and uncertain exposure–outcome linkage (Nenci, 2003).
- Decisive evidence: within-patient, time-varying PK/PD studies embedded in care pathways, analyzed using competing-risk methods (Hernán et al., 2022).
- Pregnancy and peripartum care.
- Uncertain: dose–exposure–outcome relationships across gestation and how peripartum holds and restarts affect bleeding and recurrence (Bistervels et al., 2022).
- Bias: extrapolation from non-pregnant adults and routine anti-Xa titration despite heterogeneous supporting evidence (Kjaergaard et al., 2021).
- Decisive evidence: pragmatic maternity-pathway trials that couple peripartum execution rules with exposure sampling and maternal outcomes (Bistervels et al., 2022; Kjaergaard et al., 2021).
- Cancer subtypes and high-bleeding-risk phenotypes.
- Uncertain: where LMWH retains a defensible advantage in gastrointestinal/genitourinary tumors, thrombocytopenia, or mucosal lesions, and how to operationalize phenotype-based selection (Farge et al., 2022).
- Bias: persistent “DOAC-first” versus “LMWH-safer” camps because bleeding phenotypes are not encoded into auditable decision rules (Farge et al., 2022).
- Decisive evidence: risk models integrating phenotype with biomarkers (e.g., circulating tumor deoxyribonucleic acid-informed prediction) and prospective validation of net benefit (Jee et al., 2024; Ma et al., 2025).

### 8.2 Methodological challenges

Endpoint heterogeneity (definitions of recurrent VTE and major bleeding; symptomatic versus screen-detected events) limits comparability and can invert net-benefit conclusions across studies with different detection intensity (Stevens et al., 2021; Farge et al., 2022). Competing risks are dominant in cancer and critical illness because death can preclude observed recurrent VTE or bleeding; naïve composite endpoints may therefore obscure whether a strategy prevents thrombosis or simply shortens time at risk (Farge et al., 2022). Observational comparisons of LMWH strategies are strongly confounded by indication, frailty, renal function, body weight, and cancer severity unless time zero and strategies are explicitly specified (UK Kidney Association, 2025; Hernán et al., 2022). Anti-Xa “target attainment” is a process metric rather than patient benefit and can mislead when assay variability or dose interruptions dominate achieved exposure (Farge et al., 2022; Nenci, 2003). Patient-centered outcomes—including injection burden, adherence, and quality of life—remain under-measured despite being key determinants of persistence and switching that drive real-world effectiveness (Stevens et al., 2021).

### 8.3 Future directions: feasible studies that are worth doing

The priority is to generate executable rules (dose, hold, switch, monitor) that can be embedded into routine order sets, validated against clinical outcomes rather than surrogate reassurance, and maintained by front-line clinical teams (Stevens et al., 2021).

- Model-informed dosing and exposure management. Develop population PK models with Bayesian updating using sparse sampling plus renal trajectory inputs, then validate against major bleeding and recurrent VTE (Nenci, 2003; UK Kidney Association, 2025). Minimum data: LMWH product, dosing times, sample time, body weight, creatinine trend, and adjudicated outcomes (Nenci, 2003). Decision output: next-dose recommendation plus pre-specified hold/switch triggers with uncertainty bounds (UK Kidney Association, 2025).
- Target trial emulation for bedside strategies. Specify target trials such as: “Among a pre-specified phenotype initiating therapeutic LMWH at time zero, compare fixed dosing, renal-adjusted dosing, and early-switch strategies under explicit eligibility and follow-up” (Hernán et al., 2022). Minimum data: phenotype definition, time-varying renal function, periprocedural holds, exposure history, outcomes, and a treatment-burden patient-reported measure (UK Kidney Association, 2025; Hernán et al., 2022). Decision output: phenotype-specific strategy effects that populate defaults and define when monitoring or switching should be implemented—or de-implemented (Hernán et al., 2022).
- Pregnancy and peripartum execution science. Build on Highlow trial with pragmatic trials evaluating peripartum hold/restart rules and selective monitoring triggers embedded in maternity workflows (Bistervels et al., 2022; Kjaergaard et al., 2021). Minimum data: gestational age, delivery and anesthesia timing, dosing and holds, maternal outcomes, and patient-reported burden (Bistervels et al., 2022). Decision output: auditable peripartum algorithms specifying when monitoring changes decisions (Kjaergaard et al., 2021).
- CAT phenotyping and biomarker-enabled stratification. Integrate guideline bleeding-risk phenotypes with tumor and therapy context, then test whether circulating tumor deoxyribonucleic acid-informed prediction improves targeting of anticoagulant choice and intensity (Farge et al., 2022; Jee et al., 2024; Ma et al., 2025). Minimum data: tumor site, thrombocytopenia and mucosal lesion status, systemic therapy, biomarker outputs, and adjudicated bleeding and VTE endpoints analyzed with competing-risk methods (Farge et al., 2022; Ma et al., 2025). Decision output: a two-step rule set (bleeding gate → thrombosis tier) with explicit “LMWH-favored” phenotypes (Farge et al., 2022).
- Boundary setting versus factor XI/XIa agents. Early factor XI/XIa prevention trials motivate prospective boundary setting—defining where LMWH remains strong (e.g., pregnancy, advanced chronic kidney disease, high-bleeding-risk CAT) versus where it may retreat if lower-bleeding prevention is confirmed (Verhamme et al., 2021; Weitz et al., 2021; Gailani, 2022). Minimum data: standardized endpoints and subgroup definitions aligned to current LMWH niches (UK Kidney Association, 2025; Gailani, 2022). Decision output: boundary-guided adoption criteria and explicit “safe retreat” triggers (Gailani, 2022).

## 9 Conclusion

Low-molecular-weight heparin remains useful when oral anticoagulants are difficult to use safely because of pregnancy, cancer-associated bleeding risk, renal instability, poor absorption, drug interactions, or peri-procedural timing. Its current value lies in predictable parenteral delivery and the ability to hold, restart, or switch therapy when clinical conditions change. Clinicians should document why LMWH was chosen, reassess dose and renal function during treatment, plan interruption and restart when procedures are expected, and review adherence and switching needs during follow-up.

### 9.1 Clinical next steps

- Publish an auditable pathway bundle: (i) procedure-family hold/restart rules anchored to hemostasis-confirmed sign-off and standardized documentation fields; (ii) explicit switch triggers (LMWH↔UFH↔DOAC) linked to renal trajectory, bleeding-liability phenotype, procedures, and interaction profiles; and (iii) selective monitoring triggers with standardized sampling-time rules when anti-Xa is used.
- Embed the pathway into defaults: use standardized order sets (timing and duration prompts, contraindication capture, reassessment checkpoints) so delivery fidelity does not depend on individual memory.
- Make transitions reviewable: define stop/switch criteria and surveillance windows for discharge, pregnancy-to-postpartum handoffs, and cancer-therapy changes, including a recorded switch rationale to reduce unplanned switching.

### 9.2 Research next steps

- Study monitoring that changes management: test triggered anti-Xa strategies (with sampling-time discipline) versus usual care in renal dysfunction, obesity, and non-stationary ICU physiology, alongside model-informed dosing supported by sparse sampling.
- Emulate target trials for bedside strategies: specify explicit eligibility and switching rules to compare dosing, monitoring, and LMWH↔DOAC strategies in cancer-associated thrombosis and periprocedural pathways, separating trial efficacy from system effectiveness.
- Measure burden and function, not only events: integrate treatment burden, acceptability, and longer-horizon functional outcomes alongside recurrent VTE and bleeding endpoints.

Precision dosing is clinically meaningful only when it helps clinicians make safer decisions about dose selection, monitoring, treatment interruption, restart, or switching.
