# Supplementary material for: Revisiting low-molecular-weight heparin for venous thromboembolism: from pharmacology to precision dosing and implementation
Source: Front Pharmacol. 2026 Jun 5;17:1824218. doi: 10.3389/fphar.2026.1824218 (PMC13279413; doi:10.3389/fphar.2026.1824218)
Supplement: Supplementary file 1 [file Supplementaryfile1.docx]

**Supplementary Supplementary Methods. Representative search strategy**

| **Concept** | **Representative terms** |
| --- | --- |
| LMWH | “low molecular weight heparin” OR LMWH OR enoxaparin OR dalteparin OR tinzaparin OR nadroparin |
| VTE | “venous thromboembolism” OR VTE OR “deep vein thrombosis” OR DVT OR “pulmonary embolism” OR PE |
| Dosing and monitoring | dosing OR dose adjustment OR pharmacokinetics OR pharmacodynamics OR anti-Xa OR “anti-factor Xa” OR monitoring OR accumulation OR “peak anti-Xa” |
| Special populations | renal impairment OR chronic kidney disease OR acute kidney injury OR obesity OR “extreme body weight” OR “low body weight” OR pregnancy OR postpartum OR cancer OR “cancer-associated thrombosis” OR critical illness |
| Peri-procedural management | perioperative OR periprocedural OR bridging OR interruption OR hold OR restart OR switching |
| Safety | bleeding OR reversal OR protamine OR “heparin-induced thrombocytopenia” OR HIT |

Note: Search terms were adapted for each database using controlled vocabulary where available, including MeSH terms in PubMed and Emtree terms in Embase. The table shows representative concepts rather than the complete syntax for every database.
