# Supplementary material for: Revisiting low-molecular-weight heparin for venous thromboembolism: from pharmacology to precision dosing and implementation
Source: Front Pharmacol. 2026 Jun 5;17:1824218. doi: 10.3389/fphar.2026.1824218 (PMC13279413; doi:10.3389/fphar.2026.1824218)
Supplement: Supplementary file 2 [file Table1.docx]

**Supplementary Table S1. Anti-Xa sampling and laboratory comparability checklist**

| **Domain** | **Checklist item** | **Required fields (recommended as structured EHR fields)** | **Pass criteria (comparability prerequisites)** | **Red flags / “no-go” zones (high risk of threshold transport)** | **Action if not met** |
| --- | --- | --- | --- | --- | --- |
| A. Decision preconditions | 1) Clinical question and pre-specified action | Indication for testing (exposure uncertainty / bleeding signal / procedure timing) + pre-specified action (dose/interval/switch/hold) | Question-first, test-second; action-first, interpretation-second | Testing “routinely” without a management consequence (“measurement without impact”) | Defer/cancel; manage via pathway instead |
|  | 2) Intensity context | Prophylaxis vs treatment; intended “peak” vs “trough” | Context is fixed and matches local thresholds/reporting conventions | Applying treatment thresholds to prophylaxis samples (or vice versa) | Correct context; re-sample if needed |
|  | 3) Product and dosing details | LMWH product; dose (mg or IU); route; dosing interval; timestamp of last dose | Complete documentation enabling a closed timing loop | Interpreting anti-Xa without product/dose/last-dose time | Complete fields; if unavailable, do not act |
| B. Sampling time and pre-analytics | 4) Planned sample type | Peak (local protocol “intended peak”) vs trough (pre-dose) | Sample type is selected at order entry | Trough/late samples interpreted as peak | Label as invalid for decision; re-sample per protocol |
|  | 5) Actual sampling timestamp | Draw time + minutes since last dose (Δt) | Δt is recorded; repeat testing uses consistent Δt when possible | Reviewing value without Δt; comparing results with different Δt | Require Δt display; if missing, re-sample / do not act |
|  | 6) Clock integrity | OR/ward/EHR/lab system clocks aligned | Time stamps are trustworthy | Clock drift leads to erroneous Δt | Reconcile clocks; use verified manual time if needed |
|  | 7) Collection/handling | Tube type; fill-to-mark; mixing; centrifugation; transport delay | Conforms to local SOP; key is consistency and traceability | Hemolysis/clots/prolonged delay but still interpreted | Reject and re-collect |
|  | 8) Interference and co-therapies | UFH/DOAC exposure; recent bolus; RRT/ECMO (if applicable) | Confounders documented | Unrecorded UFH exposure or recent bolus | Exclude interference; consider alternative metrics/pathway |
| C. Assay and calibration (comparability core) | 9) Assay method/platform | Chromogenic anti-Xa? instrument platform | Serial interpretation stays within the same method family | Direct comparison across different methods/platforms | Do not compare; re-establish baseline locally |
|  | 10) Calibrator strategy | Calibrator type (generic vs product-specific); source; lot (optional) | Threshold interpretation is bound to local calibration strategy | Importing “targets” from another lab/guideline/trial into a different calibration context | Prohibit threshold transport; re-interpret within local method |
|  | 11) Reagent/lot changes | Reagent brand/lot or documented change notice | Changes trigger alerts and (where applicable) re-validation | Post-change results interpreted as if unchanged | Trigger method-change note; consider repeat/verification |
|  | 12) Units and report format | Units (e.g., IU/mL); reference/decision interval; mandatory Δt display | Unit-consistent + report includes Δt and product | Unit mismatches or reports lacking Δt; forced cross-unit comparisons | Standardize reporting template; annotate legacy results as non-comparable |
| D. Interpretation and “no-threshold-transport” zones | 13) Threshold provenance | Which local SOP/pathway defines the decision interval (prophylaxis vs treatment; special populations) | Thresholds are locally traceable and context-specific | Using external thresholds (other hospital, paper, guideline) to dose-adjust locally | Require source; if absent, no dose action permitted |
|  | 14) Inter-hospital transfer rule | After lab change/transfer: baseline re-test under the new lab’s method with matched protocol/Δt | Lab change = default non-comparability; new baseline required | Using Hospital A value against Hospital B threshold to change dose | Re-test locally; actions based on the receiving lab method |
|  | 15) Longitudinal consistency | Across serial tests: same Δt, product, method | “Trend” statements only allowed if comparability holds | Declaring “rising/falling trend” despite Δt/product/method changes | Do not report trend; rebuild a consistent series |
| E. Action linkage and closure | 16) Result-to-action closure | Actual management action taken (dose/interval/hold/switch) + rationale | Action-linked interpretation (interpretation must map to an action) | Values recorded without documented action or rationale | Require action field completion; otherwise classify as non-actionable monitoring |
|  | 17) Escalation / review triggers | When to involve pharmacy/anticoagulation team/lab medicine | Triggers are explicit | High-uncertainty phenotypes without a review mechanism | Embed consult triggers in pathway |
|  | 18) QC context (minimum) | Internal QC / EQA participation (Yes/No/Unknown) | QC framework can be stated | Claiming universal thresholds without QC context | State “non-transferability” explicitly; avoid universal targets |

**Note:**

1. This checklist is designed to reduce false precision: anti-Xa is best treated as an exposure check; interpretability depends on sampling time (Δt) and local assay calibration context. Accordingly, numeric targets are not inherently transferable across laboratories, platforms, calibrators, or reporting conventions.
2. If any key fields are missing (product/dose/last-dose time/Δt/calibrator strategy/units), the result should be labeled “not valid for dose decision-making” and repeated under the local protocol.
3. The table summarizes published evidence and publicly available guidance and should not be interpreted as original unpublished clinical pathway data.

**Key sources:** Anti-Xa interpretation depends on sampling time and assay/calibrator context and is best treated as an exposure check rather than a validated efficacy surrogate; thresholds should not be transported across laboratories without local standardization. [12,13,15,16]

**Abbreviations:** anti-Xa, anti–factor Xa activity; LMWH, low-molecular-weight heparin; UFH, unfractionated heparin; VTE, venous thromboembolism; Δt, time from last dose to sampling; EHR, electronic health record; QC, quality control; EQA, external quality assessment; RRT, renal replacement therapy; ECMO, extracorporeal membrane oxygenation.
