# Supplementary material for: Revisiting low-molecular-weight heparin for venous thromboembolism: from pharmacology to precision dosing and implementation
Source: Front Pharmacol. 2026 Jun 5;17:1824218. doi: 10.3389/fphar.2026.1824218 (PMC13279413; doi:10.3389/fphar.2026.1824218)
Supplement: Supplementary file 3 [file Table2.docx]

**Supplementary Table S2. Anti-Xa monitoring triggers: when to consider, how to sample, what to do with the result**

| **Trigger (consider only if actionable)** | **Rationale (why measurement may help)** | **Sampling rule (minimum standard)** | **Action plan (pre-specify before ordering)** |
| --- | --- | --- | --- |
| Severe renal impairment or rapidly changing renal function | Accumulation/exposure drift risk as renal trajectory changes | Sample at steady state with fixed post-dose timing; document dose/interval/time precisely | Predefine: dose/interval adjust vs switch to UFH/alternative when accumulation is plausible and clinically concordant |
| Extremes of body weight (very high or very low) | Dosing heuristics may misfit; absorption/exposure uncertainty | Fixed post-dose timing; avoid “random” samples | Adjust only if result is consistent with bleeding/thrombosis phenotype; avoid reactive micro-titration |
| Pregnancy (selected high-stakes cases) | Gestational PK shifts; peripartum window is high consequence | Fixed timing; pair with peri-delivery plan; avoid ad hoc testing | Use only with explicit thresholds + a delivery/neuraxial coordination plan; consider UFH if rapid on/off needed |
| ICU/severe inflammation/marked physiologic volatility | PK instability; exposure may be unpredictable | Fixed timing; consider confirmatory repeat rather than single value | Prefer using results to support switching strategy (often UFH) rather than chasing numeric targets |
| Unexplained bleeding or recurrence on apparently appropriate dosing | Helps assess whether exposure plausibly excessive/insufficient vs non-exposure causes | Verify dosing/timing/adherence first; then fixed timing sample | If discordant with clinical picture, do not “escalate by number”; reassess diagnosis, adherence, interactions, procedures |

**Table note.** This table standardizes decision-relevant triggers for anti-Xa activity testing in patients receiving LMWH, emphasizing that monitoring is not routine and should be reserved for scenarios where results are expected to change management. Triggers are framed around physiologic volatility or dosing uncertainty (e.g., severe or rapidly changing renal function, extremes of body weight, high-stakes pregnancy/peripartum planning, or ICU-level instability) rather than blanket indications. To reduce interpretive error, sampling must be protocolized (document exact dose, dosing interval, and post-dose timing; avoid non-standard “random” samples), and teams should pre-specify how “higher/lower than expected” results will translate into an action (dose/interval adjustment, enhanced clinical surveillance, or switching strategy), interpreted alongside the bleeding/thrombosis phenotype. The goal is to avoid “number-chasing” and false precision by linking measurement to an explicit decision pathway and by prioritizing clinical concordance over isolated laboratory values. The goal is to avoid “number-chasing” and false precision by linking measurement to an explicit decision pathway and by prioritizing clinical concordance over isolated laboratory values [15,17,30,45,54].

**Abbreviations:** anti-Xa, anti-factor Xa activity; ICU, intensive care unit; LMWH, low-molecular-weight heparin; UFH, unfractionated heparin; PK, pharmacokinetics.
