# Supplementary material for: Revisiting low-molecular-weight heparin for venous thromboembolism: from pharmacology to precision dosing and implementation
Source: Front Pharmacol. 2026 Jun 5;17:1824218. doi: 10.3389/fphar.2026.1824218 (PMC13279413; doi:10.3389/fphar.2026.1824218)
Supplement: Supplementary file 4 [file Table3.docx]

**Supplementary Table S3. Expanded scenario-by-decision matrix for LMWH in VTE prevention and treatment**

| **Setting / scenario** | **Who (eligibility trigger)** | **Prevention vs treatment** | **Dose / timing / duration** | **Monitor (anti-Xa and other labs)** | **Switch / alternatives (boundary triggers)** | **Key uncertainties / implementation notes** |
| --- | --- | --- | --- | --- | --- | --- |
| **Medical inpatients (prophylaxis)** | Elevated VTE risk without contraindications; reassess as status evolves and bleeding risk changes | Prevention only; treatment pathways if confirmed VTE during stay | Start when eligible; continue through hospitalization or high-risk immobility window; avoid “more intensity = better” assumption | Anti-Xa selective only if renal function fluctuates or extreme body size; interpret as exposure check not efficacy surrogate | Mechanical only if bleeding risk dominates; UFH if need rapid on/off or severe renal impairment; DOAC rare in acute medical ward setting | Delivery fidelity (order sets, contraindication capture) and daily reassessment drive effect more than agent choice |
| **Surgical / peri-operative (prophylaxis)** | Procedure family risk × patient risk; mechanical layering when VTE risk extreme | Prevention; switch to treatment only if post-op VTE occurs [Stevens SM] | Start after hemostasis-confirmed “safe start”; duration per procedure type (orthopedic / oncologic often longer) | Routine anti-Xa not indicated; selective only for extreme PK uncertainty and protocol-linked actions | Mechanical only if prohibitive bleeding; UFH transiently when unstable or titration needed | Operational definition of “adequate hemostasis” and sign-off responsibility remain variable locally |
| **Acute VTE (non-cancer)** | Need for parenteral therapy when oral not yet feasible or bridging required | Treatment of established VTE [Stevens SM] | Therapeutic dosing per indication; protocolized holds around procedures; reassess with renal changes | Selective anti-Xa if renal fluctuation, extreme weight, ICU non-stationary PK, or unexpected signal | Transition to DOAC or warfarin when stable; UFH preferred if fine titration or rapid on/off required | Need clear criteria for when to switch from parenteral to oral and document timing triggers |
| **Cancer-associated thrombosis (CAT)** | High bleeding-liability phenotypes; complex drug interactions; unreliable absorption; recent procedures | Treatment of established CAT | Continue while cancer active / therapy ongoing; reassess at procedures, platelet drops, regimen changes | Anti-Xa used only as exposure check in high-uncertainty contexts; focus on bleeding surveillance | DOACs reasonable in selected lower-bleeding-risk phenotypes; LMWH preferred when uncertainty dominates; UFH peri-procedure if unstable | Real-world switching frequent; trial efficacy vs system effectiveness diverge |
| **Pregnancy / postpartum** | Risk-stratified prophylaxis; LMWH central for acute VTE treatment | Prevention and treatment per stage; reassess around delivery | Antepartum + postpartum windows; peri-delivery hold/restart rules; extend if persistent risk | Anti-Xa selective only for extreme weight or renal dysfunction where result changes dose | UFH near delivery for rapid on/off; mechanical adjunct if bleeding risk dominates | Documentation of hold/restart timing and responsible sign-off essential |
| **Renal impairment / fluctuating renal function** | Severe impairment or rapid renal change | Either prevention or treatment depending on indication | Adjust dose or interval per protocol; frequent re-evaluation | Anti-Xa most defensible when renal function severe or fluctuating and result will change dose | UFH if titration needed or renal clearance too low | Sampling-time discipline and assay calibration critical |
| **Extremes of body size / obesity** | Very low or very high body weight; obesity with bleeding concerns | Prevention and treatment contexts as applicable | Dose selection based on protocol; reassess with clinical course | Anti-Xa selective if it will change dose; avoid “monitor for reassurance” | Switch if discordant clinical signals persist | Document dose rationale and monitoring decision pathway |
| **ICU / inflammation / ECMO / CRRT** | Vasopressors, ARC, CRRT / ECMO, rapid clinical changes | Prevention or treatment depending on indication | Prefer pathways allowing rapid re-assessment | Anti-Xa only when result changes management; interpret cautiously | UFH preferred when continuous titration or rapid interruption required | Standardize sampling and action thresholds for non-stationary PK contexts |
| **Extended prophylaxis / secondary prevention** | High recurrence-risk phenotypes; adherence issues | Prevention of recurrent VTE | Duration individualized; reassess risk trajectory | Generally no anti-Xa; focus on adherence and bleeding surveillance | DOACs often preferred when eligible; LMWH if oral not feasible | Build follow-up and re-evaluation triggers into care pathway |

**Note:** Supplementary Table S3 expands Table 2 to provide the full decision elements (who/intent/timing–duration/monitoring/switching/implementation notes) by setting, anchored to guideline statements, key trials/meta-analyses in high-impact settings (e.g., perioperative prophylaxis; CAT DOAC–LMWH boundaries), and monitoring syntheses emphasizing selective, action-linked anti-Xa use. Key sources informing the expanded matrix include: [1,8,9,11,15,20,21,23-26].

**Abbreviations:** anti-Xa, anti-factor Xa activity; ARC, augmented renal clearance; CAT, cancer-associated thrombosis; CRRT, continuous renal replacement therapy; DOAC, direct oral anticoagulant; DVT, deep vein thrombosis; ECMO, extracorporeal membrane oxygenation; GI/GU, gastrointestinal/genitourinary; ICU, intensive care unit; LMWH, low-molecular-weight heparin; PE, pulmonary embolism; PK, pharmacokinetics; UFH, unfractionated heparin; VTE, venous thromboembolism.
