# Supplementary material for: Revisiting low-molecular-weight heparin for venous thromboembolism: from pharmacology to precision dosing and implementation
Source: Front Pharmacol. 2026 Jun 5;17:1824218. doi: 10.3389/fphar.2026.1824218 (PMC13279413; doi:10.3389/fphar.2026.1824218)
Supplement: Supplementary file 5 [file Table4.docx]

**Supplementary Table S4. Switch / alternatives: triggers and implementation notes**

| **Trigger** | **Preferred alternative / strategy** | **Implementation note (make it auditable)** |
| --- | --- | --- |
| Imminent procedure or extreme bleeding volatility (need rapid on/off, titration) | UFH (or hold per pathway) | Document accountable decision-maker, escalation, and switch-back/reassessment plan |
| Oral pathway operationally fragile (nausea, unreliable absorption, interaction burden, unstable renal trajectory) | LMWH “bridge” then transition | Define an early checkpoint for transition once stabilized; ensure follow-up and interaction review |
| CAT with GI/GU bleeding-liability phenotype, luminal lesions, or high uncertainty | Prefer LMWH; UFH transiently if rapid interruption needed | Treat injection burden/access as implementation variables; plan persistence supports and reassessment |
| CAT transitions (regimen change, procedure cluster, thrombocytopenia swing) | LMWH ↔ DOAC (phenotype-matched) or UFH transiently | Require documented rationale + planned reassessment; execute interaction checks and bleeding surveillance across transitions |
| Peri-delivery window with neuraxial planning / peripartum volatility | UFH (or hold) per pathway; resume LMWH once hemostasis acceptable | Pre-specify hold/restart rules and coordinate with anesthesia; document timing and responsibility |
| Injection burden threatens adherence, or preference dominates (and bleeding risk acceptable) | Transition to DOAC/VKA when feasible | Treat burden as a pathway variable; ensure education, access, and follow-up checkpoints |
| Extended/secondary prevention reassessment after initial window | Full-dose vs reduced-intensity vs stop (often oral reduced-intensity when feasible) | Record the chosen lane and reassessment date; communicate residual risk and revisit with patient priorities |

**Table note.** This table consolidates practical triggers for deviating from an LMWH pathway and selecting common alternatives—UFH, DOACs, or VKAs—with brief implementation notes to support auditable bedside decisions. Triggers emphasize operational feasibility and safety constraints (e.g., need for rapid on/off around procedures or delivery, evolving bleeding risk, high drug–drug interaction burden, unreliable gastrointestinal absorption, unstable renal trajectory, injection burden affecting adherence, and postpartum transition feasibility). “Switch” implies a planned transition with documented timing, follow-up checkpoints (renal function, bleeding, adherence), and a clear reassessment or “switch-back” plan when used to navigate short-term volatility. The table is not an exhaustive list of indications or contraindications; it is a structured prompt to standardize common decision points and reduce unwarranted practice variation while preserving clinician judgment and patient preference. The synthesis draws on contemporary international guidelines and cross-setting evidence for peri-procedural management, cancer-associated thrombosis, pregnancy-related pathways, and extended/secondary prevention [7,8,10,23,39,42,49].

**Abbreviations:** CAT, cancer-associated thrombosis; DOAC, direct oral anticoagulant; GI, gastrointestinal; GU, genitourinary; LMWH, low-molecular-weight heparin; UFH, unfractionated heparin; VKA, vitamin K antagonist.
